# Editorial: Role of the Thalamus in Motivated Behavior

**DOI:** 10.3389/fnbeh.2021.720592

**Published:** 2021-07-02

**Authors:** Morgan H. James, Gavan P. McNally, Xuan Li

**Affiliations:** ^1^Department of Psychiatry, Robert Wood Johnson Medical School and Rutgers Biomedical Health Sciences, Rutgers University, Piscataway, NJ, United States; ^2^Brain Health Institute, Rutgers Biomedical Health Sciences, Rutgers University, Piscataway, NJ, United States; ^3^School of Psychology, University of New South Wales Sydney, Sydney, NSW, Australia; ^4^Department of Psychology, University of Maryland, College Park, MD, United States

**Keywords:** cortico-striatal-thalamic-cortical circuit, motivated behavior, paraventricular thalamus, thalamostriatal circuit, learning, memory, decision making, addiction

Growing evidence shows that the thalamus, beyond serving as an information relaying center, has key roles in motivated behaviors (Martin-Fardon and Boutrel, 2012; James and Dayas, 2013; Kirouac, 2015; Millan et al., 2017; Huang et al., 2018; Choi et al., 2019; Otis et al., 2019; McNally, 2021). The aim of this Research Topic is to highlight the specific roles of distinct thalamic nuclei in a variety of motivated behaviors. Our collection of 10 articles includes four reviews, one mini-review, one perspective, one hypothesis-and-theory, and three original research papers. Among these, the majority focus on paraventricular thalamic nucleus (PVT, a midline thalamic nucleus), while others highlight rostral intralaminar thalamic nuclei (rILN), posterior intralaminar thalamic nuclei (also known as parafascicular thalamic nuclei, Pf), and mediodorsal thalamus (MD, another midline thalamic nucleus). Taken together, this collection provides evidence that thalamus integrates and processes information within the cortico-striatal-thalamo-cortical circuit to guide salience processing, adaptive controls, cognitive engagement, feeding and drug seeking.

The hypothesis-and-theory by Worden et al. proposes that thalamus functions as a central blackboard in cognition with an emphasis on three distinct thalamic nuclei: pulvinar, MD, and PVT. These nuclei, through their anatomical connections with cortical and other thalamic regions, entrain the cortico-cortical circuitry to take over routine tasks and therefore spare thalamus for engagement in novel tasks. Although empirical data directly corroborating these intriguing views are not yet available, a role of thalamus and its associated circuitry in cognitive and emotional processes is well-documented. The mini-review by Zhou et al. summarizes recent findings on thalamic circuits implicated in reward, pain processing, arousal, attention controls, and adaptive behavior. These thalamic activities (especially those associated with PVT) contribute to both normal (e.g., associative learning) and abnormal (e.g., drug addiction, posttraumatic stress disorder and schizophrenia) salience processing.

In our collection, five articles exclusively focus on PVT. The original research article by Quiñones-Laracuente et al. examined the time-dependent recruitment of pre-limbic (PL) prefrontal inputs onto PVT following auditory fear learning. The authors showed that PL to PVT projections are activated by conditioned stimuli (CS) 7 d, but not 2 h, following learning. In contrast, the PL-amygdala circuit is preferentially recruited 2 h following learning. In addition, unit recordings of Layer VI PL neurons, the origin of projections to PVT, exhibit increased cue-induced inhibition at later, but not earlier, time points. Together, these results suggest that PL signaling of simple fear associations shifts with time toward inhibitory modulation of PVT, which may underlie disinhibition of PVT neurons (via neurons in reticular nucleus of thalamus) and subsequently enhanced central amygdala output.

The original research article by Matzeu and Martin-Fardon reported that posterior PVT injections of orexin-A peptide promotes reinstatement of extinguished cocaine seeking after intermediate (2–3 weeks), but not protracted (4–5 weeks), abstinence. Intermediate but not protracted abstinence is associated with an upregulation of orexin 2 receptor expression in PVT, while orexin cell numbers increase after both intermediate and protracted abstinence. This work extends previous work on the role of hypothalamic orexin (hypocretin) neurons in PVT in addiction-related behavior (Dayas et al., 2008; Mahler et al., 2014; Matzeu et al., 2016; Ubaldi et al., 2016; James et al., 2017; Matzeu and Martin-Fardon, 2020), and supports emerging evidence linking increased orexin signaling to addiction propensity (Thannickal et al., 2018; Fragale et al., 2019; James et al., 2019; Collier et al., 2020; Pantazis et al., 2020).

Munkhzaya et al. recorded PVT unit activities in rats performing a cue-licking task to determine the involvement of PVT in the predictive vs. incentive information of CS. Neural activity in PVT immediately after CS onset discriminates reward/non-reward association (predictive information) but not reward value (incentive information). In contrast, activity of PVT neurons that fire immediately before reward delivery is correlated with reward value but not predictive information. Together, these data capture the heterogeneity of PVT responses to discrete processes involved in cue-induced motivated behaviors.

PVT is also a regulator of stress (Beas et al., 2018; Dong et al., 2020). Rowson and Pliel provide a timely review on the sex-dependent effects of acute vs. chronic stress on PVT, and outline the implications of this dimorphism for motivated behaviors. Consistent with the idea of PVT as a complex integrator of varied physiological signals, Petrovich elegantly discusses the role of PVT in controling feeding behavior. Petrovich describes a framework whereby PVT integrates homeostatic and hedonic needs to feed with physiological and environmental stress signals, ultimately guiding the balance between food seeking and consumption.

Two reviews focus on ILN, recently implicated in goal-direct behaviors (Bradfield et al., 2013; Bradfield and Balleine, 2017; Li et al., 2018; Cover et al., 2019). Cover and Mathur reveal distinct anatomic, physiologic, and synaptic properties of rILN through comparison with other thalamic nuclei, such as thalamocortical relay nuclei and the Pf. Together with evidence implicating rILN in arousal, pain, executive function, and action control, the authors propose a unique role of rILN in task-dependent behavioral engagement, such as goal valuation based on interceptive and external factors, action learning, expression and reinforcement. Stayte et al. review the function of Pf and orbitofrontal cortex (OFC) in action selection. Further discussion of afferents of each structure leads to the hypothesis that Pf and OFC together contribute to internal state representation during action selection either through direct Pf to OFC projections or convergence of their respective inputs onto striatal cholinergic interneurons.

Finally, Mair et al. discuss the roles of medial prefrontal cortex (mPFC) and individual central thalamic nuclei (e.g., PVT, rILN, and MD) in delayed conditional discrimination tasks through lesion studies in rodents. The authors review electrophysiological findings in MD and mPFC during adaptive goal-directed behaviors, which suggest that MD affects both action and outcome-related neuronal responses in mPFC.

We appreciate these excellent contributions. These articles not only summarize the current findings on the role of individual thalamic nuclei mediating motivated behavior, but also raise intriguing questions about how thalamus exerts these effects. We hope that this issue gives impetus to ongoing work in the field to better characterize the role of thalamus in motivated behaviors and related disorders.

## Author Contributions

XL and MJ took the lead in writing this editorial. GM contributed to its finalization. All authors contributed to the article and approved the submitted version.

## Conflict of Interest

The authors declare that the research was conducted in the absence of any commercial or financial relationships that could be construed as a potential conflict of interest.

## References

[B1] Beas B. S. Wright B. J. Skirzewski M. Leng Y. Hyun J. H. Koita O.. (2018). The locus coeruleus drives disinhibition in the midline thalamus via a dopaminergic mechanism. Nat. Neurosci. 21, 963–973. 10.1038/s41593-018-0167-4 29915192 PMC6035776

[B2] Bradfield L. A. Balleine B. W. (2017). Thalamic control of dorsomedial striatum regulates internal state to guide goal-directed action selection. J. Neurosci. 37, 3721–3733. 10.1523/JNEUROSCI.3860-16.2017 28242795 PMC6596916

[B3] Bradfield L. A. Bertran-Gonzalez J. Chieng B. Balleine B. W. (2013). The thalamostriatal pathway and cholinergic control of goal-directed action: interlacing new with existing learning in the striatum. Neuron 79, 153–166. 10.1016/j.neuron.2013.04.039 23770257 PMC3863609

[B4] Choi E. A. Jean-Richard-Dit-Bressel P. Clifford C. W. G. McNally G. P. (2019). Paraventricular thalamus controls behavior during motivational conflict. J. Neurosci. 39, 4945–4958. 10.1523/JNEUROSCI.2480-18.2019 30979815 PMC6670259

[B5] Collier A. D. Min S. S. Campbell S. D. Roberts M. Y. Camidge K. Leibowitz S. F. (2020). Maternal ethanol consumption before paternal fertilization: stimulation of hypocretin neurogenesis and ethanol intake in zebrafish offspring. Prog. Neuropsychopharmacol. Biol. Psychiatry 96:109728. 10.1016/j.pnpbp.2019.109728 31394141 PMC6815720

[B6] Cover K. K. Gyawali U. Kerkhoff W. G. Patton M. H. Mu C. White M. G.. (2019). Activation of the rostral intralaminar thalamus drives reinforcement through striatal dopamine release. Cell Rep. 26, 1389–1398. 10.1016/j.celrep.2019.01.044 30726725 PMC6402336

[B7] Dayas C. V. McGranahan T. M. Martin-Fardon R. Weiss F. (2008). Stimuli linked to ethanol availability activate hypothalamic CART and orexin neurons in a reinstatement model of relapse. Biol. Psychiatry 63, 152–157. 10.1016/j.biopsych.2007.02.002 17570346

[B8] Dong X. Li S. Kirouac G. J. (2020). A projection from the paraventricular nucleus of the thalamus to the shell of the nucleus accumbens contributes to footshock stress-induced social avoidance. Neurobiol. Stress 13:100266. 10.1016/j.ynstr.2020.100266 33344719 PMC7739169

[B9] Fragale J. E. Pantazis C. B. James M. H. Aston-Jones G. (2019). The role of orexin-1 receptor signaling in demand for the opioid fentanyl. Neuropsychopharmacology 44, 1690–1697. 10.1038/s41386-019-0420-x 31112988 PMC6785092

[B10] Huang A. S. Mitchell J. A. Haber S. N. Alia-Klein N. Goldstein R. Z. (2018). The thalamus in drug addiction: from rodents to humans. Philos. Trans. R. Soc. Lond. B Biol. Sci. 373:201700028. 10.1098/rstb.2017.0028 29352027 PMC5790826

[B11] James M. H. Dayas C. V. (2013). What about me.? The PVT: a role for the paraventricular thalamus (PVT) in drug-seeking behavior. Front. Behav. Neurosci. 7:18. 10.3389/fnbeh.2013.00018 23509439 PMC3589664

[B12] James M. H. Mahler S. V. Moorman D. E. Aston-Jones G. (2017). A decade of orexin/hypocretin and addiction: where are we now? Curr. Top. Behav. Neurosci. 33, 247–281. 10.1007/7854_2016_57 28012090 PMC5799809

[B13] James M. H. Stopper C. M. Zimmer B. A. Koll N. E. Bowrey H. E. Aston-Jones G. (2019). Increased number and activity of a lateral subpopulation of hypothalamic orexin/hypocretin neurons underlies the expression of an addicted state in rats. Biol. Psychiatry 85, 925–935. 10.1016/j.biopsych.2018.07.022 30219208 PMC7528037

[B14] Kirouac G. J. (2015). Placing the paraventricular nucleus of the thalamus within the brain circuits that control behavior. Neurosci. Biobehav. Rev. 56, 315–329. 10.1016/j.neubiorev.2015.08.005 26255593

[B15] Li X. Witonsky K. R. Lofaro O. M. Surjono F. Zhang J. Bossert J. M.. (2018). Role of anterior intralaminar nuclei of thalamus projections to dorsomedial striatum in incubation of methamphetamine craving. J. Neurosci. 38, 2270–2282. 10.1523/JNEUROSCI.2873-17.2018 29371321 PMC5830515

[B16] Mahler S. V. Moorman D. E. Smith R. J. James M. H. Aston-Jones G. (2014). Motivational activation: a unifying hypothesis of orexin/hypocretin function. Nat. Neurosci. 17, 1298–1303. 10.1038/nn.3810 25254979 PMC4335648

[B17] Martin-Fardon R. Boutrel B. (2012). Orexin/hypocretin (Orx/Hcrt) transmission and drug-seeking behavior: is the paraventricular nucleus of the thalamus (PVT) part of the drug seeking circuitry? Front. Behav. Neurosci. 6:75. 10.3389/fnbeh.2012.00075 23162448 PMC3494007

[B18] Matzeu A. Kerr T. M. Weiss F. Martin-Fardon R. (2016). Orexin-A/Hypocretin-1 mediates cocaine-seeking behavior in the posterior paraventricular nucleus of the thalamus via orexin/hypocretin receptor-2. J. Pharmacol. Exp. Ther. 359, 273–279. 10.1124/jpet.116.235945 27540003 PMC5074483

[B19] Matzeu A. Martin-Fardon R. (2020). Targeting the orexin system for prescription opioid use disorder: orexin-1 receptor blockade prevents oxycodone taking and seeking in rats. Neuropharmacology 164:107906. 10.1016/j.neuropharm.2019.107906 31841797 PMC6954966

[B20] McNally G. P. (2021). Motivational competition and the paraventricular thalamus. Neurosci. Biobehav. Rev. 125, 193–207. 10.1016/j.neubiorev.2021.02.021 33609570

[B21] Millan E. Z. Ong Z. McNally G. P. (2017). Paraventricular thalamus: gateway to feeding, appetitive motivation, and drug addiction. Prog. Brain Res. 235, 113–137. 10.1016/bs.pbr.2017.07.006 29054285

[B22] Otis J. M. Zhu M. Namboodiri V. M. K. Cook C. A. Kosyk O. Matan A. M.. (2019). Paraventricular thalamus projection neurons integrate cortical and hypothalamic signals for cue-reward processing. Neuron 103, 423–431.e424. 10.1016/j.neuron.2019.05.018 31196673 PMC6773659

[B23] Pantazis C. B. James M. H. Bentzley B. S. Aston-Jones G. (2020). The number of lateral hypothalamus orexin/hypocretin neurons contributes to individual differences in cocaine demand. Addict. Biol. 25:e12795. 10.1111/adb.12795 31297913 PMC7083199

[B24] Thannickal T. C. John J. Shan L. Swaab D. F. Wu M. F. Ramanathan L.. (2018). Opiates increase the number of hypocretin-producing cells in human and mouse brain and reverse cataplexy in a mouse model of narcolepsy. Sci. Transl. Med. 10:eaao4953. 10.1126/scitranslmed.aao4953 29950444 PMC8235614

[B25] Ubaldi M. Giordano A. Severi I. Li H. Kallupi M. de Guglielmo G.. (2016). Activation of hypocretin-1/orexin-A neurons projecting to the bed nucleus of the stria terminalis and paraventricular nucleus is critical for reinstatement of alcohol seeking by neuropeptide S. Biol. Psychiatry 79, 452–462. 10.1016/j.biopsych.2015.04.021 26055195

